# Reorganization of a synthetic microbial consortium for one-step vitamin C fermentation

**DOI:** 10.1186/s12934-016-0418-6

**Published:** 2016-01-25

**Authors:** En-Xu Wang, Ming-Zhu Ding, Qian Ma, Xiu-Tao Dong, Ying-Jin Yuan

**Affiliations:** Key Laboratory of Systems Bioengineering (Ministry of Education), School of Chemical Engineering and Technology, Tianjin University, Tianjin, 300072 People’s Republic of China; SynBio Research Platform, Collaborative Innovation Center of Chemical Science and Engineering (Tianjin), Tianjin University, Tianjin, 300072 People’s Republic of China

**Keywords:** Synthetic microbial consortium, Reorganization, One-step fermentation, Interaction, Metabolomics

## Abstract

**Background:**

In the industry, the conventional two-step fermentation method was used to produce 2-keto-l-gulonic acid (2-KGA), the precursor of vitamin C, by three strains, namely, *Gluconobacter oxydans*, *Bacillus* spp. and *Ketogulonicigenium vulgare*. Despite its high production efficiency, the long incubation period and an additional second sterilization process inhibit the further development. Therefore, we aimed to reorganize a synthetic consortium of *G. oxydans* and *K. vulgare* for one-step fermentation of 2-KGA and enhance the symbiotic interaction between microorganisms to perform better.

**Results:**

During the fermentation, competition for sorbose of *G. oxydans* arose when co-cultured with *K. vulgare*. In this study, the competition between the two microbes was alleviated and their mutualism was enhanced by deleting genes involved in sorbose metabolism of *G. oxydans*. In the engineered synthetic consortium (H_6_ + Kv), the yield of 2-KGA (mol/mol) against d-sorbitol reached 89.7 % within 36 h, increased by 29.6 %. Furthermore, metabolomic analysis was used to verify the enhancement of the symbiotic relationship and to provide us potential strategies for improving the synthetic consortium. Additionally, a significant redistribution of metabolism occurred by co-culturing the *K. vulgare* with the engineered *G. oxydans*, mainly reflected in the increased TCA cycle, purine, and fatty acid metabolism.

**Conclusions:**

We reorganized and optimized a synthetic consortium of *G. oxydans* and *K. vulgare* to produce 2-KGA directly from d-sorbitol. The yield of 2-KGA was comparable to that of the conventional two-step fermentation. The metabolic interaction between the strains was further investigated by metabolomics, which verified the enhancement of the mutualism between the microbes and gave us a better understanding of the synthetic consortium.

**Electronic supplementary material:**

The online version of this article (doi:10.1186/s12934-016-0418-6) contains supplementary material, which is available to authorized users.

## Background

As synthetic biology begins to address problems involved in the programing novel biological systems, engineering multicellular behavior is emerging as a key tool for building advanced synthetic systems that robustly perform complex behaviors [1]. In natural environments, microorganisms commonly exist as communities of multiple species that are capable of fulfilling more varied and complicate tasks than clonal populations [2]. Different members of a consortium assume different responsibilities, increasing overall productivity and allowing for more complex behavior than that with a single cell or a monoculture. During the last decade, experimental efforts have been made to build and maintain the synthetic communities [3–5]. However, those studies were mostly concerned with the well-defined ideal models (e.g., mutualism, parasitism and commensalism, etc.). Whereas in industrially-relevant circumstances, the situation is more complex since the exchange of metabolites, energy, and informative signals with the environment should be taken into consideration, and the relationship among the strains is often too diverse to analyze. Therefore, increasing attention has been paid on the development of synthetic consortia in industry. Several microbial consortia were constructed for studying the cooperation of cells, enhancing the production of biofuel [6, 7], electricity [8] and even complex natural products [9], etc.

Generally, microorganisms interact with each other by exchanging biomolecules (e.g., proteins, nucleic acids and metabolites) and information signals via contact-based or contact-independent interaction [10]. Omics study can provide deep insights into the mechanism of metabolic crosstalk in a consortium at the global level and indicate the way to better understand the relationship between the species [11]. Researchers have adopted this approach to investigate the co-cultured microbial systems, including some previous studies about the metabolic cooperation of *Bacillus megaterium* and *Ketogulonicigenium vulgare* in two-step vitamin C fermentation [12–16]. In this study, metabolomic analysis was used to better understand the specialization and cooperation between *Gluconobacter oxydans* and *K. vulgare* in the reorganized microbial consortium. These analyses verified the alleviation of competition and the enhancement of the symbiotic relationship, which provided us potential strategies for further construction of the microbes.

Chen et al. [17] declared the fundamental power of cell specialization and cooperation in the consortia. It is a powerful reminder that the communities are frequently more than the sum of their parts. In nature, microbes can form interacting communities to accomplish chemically difficult tasks through division of labor among different species [18]. In this study, the industrial vitamin C fermentation was taken as an example. The conventional two-step fermentation method was used to produce 2-keto-l-gulonic acid (2-KGA), the precursor of vitamin C, by three strains (*G. oxydans*, *Bacillus* spp. and *K. vulgare*). During the second step, *K. vulgare* is responsible for the biosynthesis of 2-KGA from l-sorbose, and *Bacillus* spp., as a companion, promotes the growth and production efficiency of *K. vulgare*. Despite its high production efficiency, the long incubation period and an additional second sterilization process of the two-step fermentation inhibit the further development of industrial production. Hence, we demonstrated the concept of reconstituting a heterologous metabolic pathway in a microbial partnership with *G. oxydans* and *K. vulgare*, where 2-KGA was produced directly from d-sorbitol. Furthermore, two genes involved in sorbose metabolism from *G. oxydans* were knocked out to alleviate the competition for sorbose of *G. oxydans*. The yield of 2-KGA (mol/mol) against d-sorbitol reached 89.7 % (76.6 g/L) within 36 h, which enabled an 29.6 % increase compared to the original consortium 69.3 % (59.1 g/L). Additionally, simplifying metabolic pathway may remove some negative effects for the microbes and increase the metabolic efficiency, which makes up for the mismatch of the consortium and enhances the cell–cell interaction. Hence, metabolomic analysis was used to provide a clear and comprehensive description of the physiological relationship between them, which was the key issue of this one-step fermentation. Compared with the conventional two-step fermentation process, this new route of one-step fermentation can potentially revolutionize the industrial-scale production of vitamin C.

## Methods

### Strains

The two industrial strains, *G. oxydans* and *K. vulgare*, used in this study were generously supplied by Welcome Pharmaceutical Co., Ltd. North China Pharmaceutical Group (Shijiazhuang, China). The stepwise construction of mutant *G. oxydans* strains with target gene deletion by homologous recombination is shown in Additional file 1: Figure S1. *Escherchia coli* DH5α strain, purchased from Takara (Dalian, China), was used as an intermediate host for plasmid construction and propagation. Firstly, a promoter tufB and the recombination flanks of the target genes were individually amplified from the genome of *G. oxydans*. Then a selection marker *gen* or *tet* was amplified from two broad-host vectors. After that, all these four fragments were ligated through overlap extension PCR (OE PCR), forming gene deletion cassettes. During the whole process, all fragments of different cassettes were cloned into *E. coli* DH5α using standard methods with a cloning vector *pEASY*-Blunt (TransGene Biotech Co., Ltd. Beijing). Sequentially, we transformed the cassettes into *G. oxydans* by electroporation and selected the correct clones from the agar plates with 210 μg/ml gentamycin or 25 μg/ml tetracycline. All the engineered bacterial strains got in this study were described in Table 1.

**Table 1:** Relevant information of engineered strains in this study

Strains (abbreviations)	Target genes deleted
SyBE_Go00010308 (H_1_)	B932_0664: FAD-dependent l-sorbose 1-dehydrogenase
SyBE_Go00010309 (H_2_)	B932_1330: NADPH-dependent l-sorbose reductase
SyBE_Go00010310 (H_3_)	B932_1370: PTS system transporter subunit IIA
SyBE_Go00010311 (H_4_)	B932_1684: NADPH-dependent l-sorbose reductase
SyBE_Go00010312 (H_5_)	B932_3022: NADPH-dependent l-sorbose reductase
SyBE_Go00010313 (H_6_)	B932_1330 and B932_1370

### Medium and culture conditions

All *E. coli* strains were cultivated in Luria-Broth (LB) medium at 37 °C. The d-sorbitol/l-sorbose seed culture medium for the mono-culture of *G. oxydans* and *K. vulgare* was composed of 20 g/L d-sorbitol (for *G. oxydans*) or l-sorbose (for *K. vulgare*), 10 g/L peptone, 3 g/L corn-steep liquor (CSL), 3 g/L beef extract, 3 g/L yeast extract, 1 g/L urea, 1 g/L KH_2_PO_4_, 1 g/L CaCO_3_ and 0.2 g/L MgSO_4_·7H_2_O. The fermentation medium for the one-step co-culture contained 80 g/L d-sorbitol, 10 g/L CSL, 12 g/L urea, 1 g/L KH_2_PO_4_, 1 g/L CaCO_3_ and 0.2 g/L MgSO_4_·7H_2_O. The pH values of the medium were maintained at 7.0 by the addition of NaOH.

The mono-culture of *G. oxydans* and *K. vulgare*, as seed for the subsequent co-culture fermentation, were cultivated in 250 ml flasks with 50 ml d-sorbitol/l-sorbose seed cultures at 30 °C and 250 rpm for 24 h. The OD_600_ of *G. oxydans* and *K. vulgare* in the seed culture reached about 5.5 and 3.0, respectively. Whereafter, these two strains were simultaneously inoculated into a 5 L jar fermentor (Bailun Bio-technology Co. Ltd., Shanghai) with 3 L fermentation medium. The inoculation ratio (%, v/v) of *G. oxydans* and *K. vulgare,* agitation speed and aeration rate were optimized. The initial inoculum ratio of *G. oxydans* and *K. vulgare* was 4:1. pH value and temperature of the fed-batch fermentation were automatically controlled at 7.0 and 30 °C. And the agitation speed was controlled at 500 rpm with the aeration rate of 1.5 vvm.

### Analysis of population of each species

Co- and mono-cultured community samples were collected from fermentations at 0, 4, 8, 14, 21, 28 and 32 h after inoculation. The genomic DNA was extracted from the samples with the TIANamp Bacteria DNA Kit (Tiangen Biotech, China). RealMasterMix (SYBR Green) was used and the quantitative PCR reactions were performed on Light-Cycler 480 with the primers designed based on 16S rDNA of each species 5′-CGATGTGTGCTGGATGTTGGG-3′ and 5′-TCTGAACCGGTCCTCCCCATG-3′ for *G. oxydans*, and 5′-AATGCCAGTCGTCAGGTTGCTT-3′ and 5′-CTAGGCCGGTCCTGTAATGTCA-3′ for *K. vulgare*. The amount of genome of each species was computed by comparison with a standard curve from pure cultures analyzed with the same manner.

### Transcriptional analysis of relevant genes in co- and mono-cultured systems

The transcriptional expression level of the genes in co- and mono-cultured systems at different sampling times was evaluated by qPCR. All the data were normalized to 16S rDNA of each species. The entire RNA was extracted from the samples with the ApexPrep RNA Miniprep Kit (APExBio). HiTaq EvaGreen qPCR MasterMix (APExBio) was used and the quantitative PCR reactions were performed on a CFX96 real time PCR system (Bio-Rad) with a total volume of 20 μL containing diluted cDNA (2 μL), qPCR MasterMix (10 μL),and forward primer and reverse primer (0.8 μL).

### Sample preparation and metabolites analysis

The samples from different co- and mono-cultured fermentations were collected at 4, 8, 14, 21 and 28 h after inoculation. These five time points primarily represented the lag phase, the early exponential phase, the middle exponential phase, the late exponential phase and the stationary phase of community. The concentrations of extracellular d-sorbitol, l-sorbose and 2-KGA were analyzed by HPLC (Waters Corp., USA) with a refractive index detector. H_2_SO_4_ (5 mM) was used as the mobile phase on an Aminex HPX-87H column (BioRad, CA) at the temperature of 65 °C with a flow rate of 0.6 ml/min.

The intracellular metabolites were extracted and derivatizated according to our previous procedure [19]. Gas chromatography time-of-flight mass spectrometry (GC-TOF/MS, Waters Corp., USA) was applied to detect the metabolites in different samples, as described by Ding et al. [20] with identical chromatographic conditions. One microlitre sample was injected with a split ratio of 1:1 into GC, equipped with a fused-silica capillary column (DB-5MS, 30 m × 0.25 mm i.d., 0.25 μm, J&W Scientific, Folsom, CA, USA). After a 2 min delay at 70 °C, the oven temperature program increased to 290 °C at 5 °C/min, holding for 3 min. The temperature of the transfer line and the ion source was 280 and 250 °C, respectively. Helium (99.9995 %) was used as the carrier gas under a constant pressure of 91 kPa. The solvent delay was 5 min. Ions were generated by a 70 eV electron beam at an ionization current of 40 μA. Two spectra were recorded per second in the mass range of 50–800 m/z with DRE function.

Principal component analysis (PCA) and pathway enrichment analysis were performed by MetaboAnalyst 3.0 (http://www.metaboanalyst.ca/MetaboAnalyst/faces/home.xhtml) [21].

## Results and discussion

### Reorganization of a synthetic microbial consortium for one-step vitamin C fermentation

**Fig. 1:**
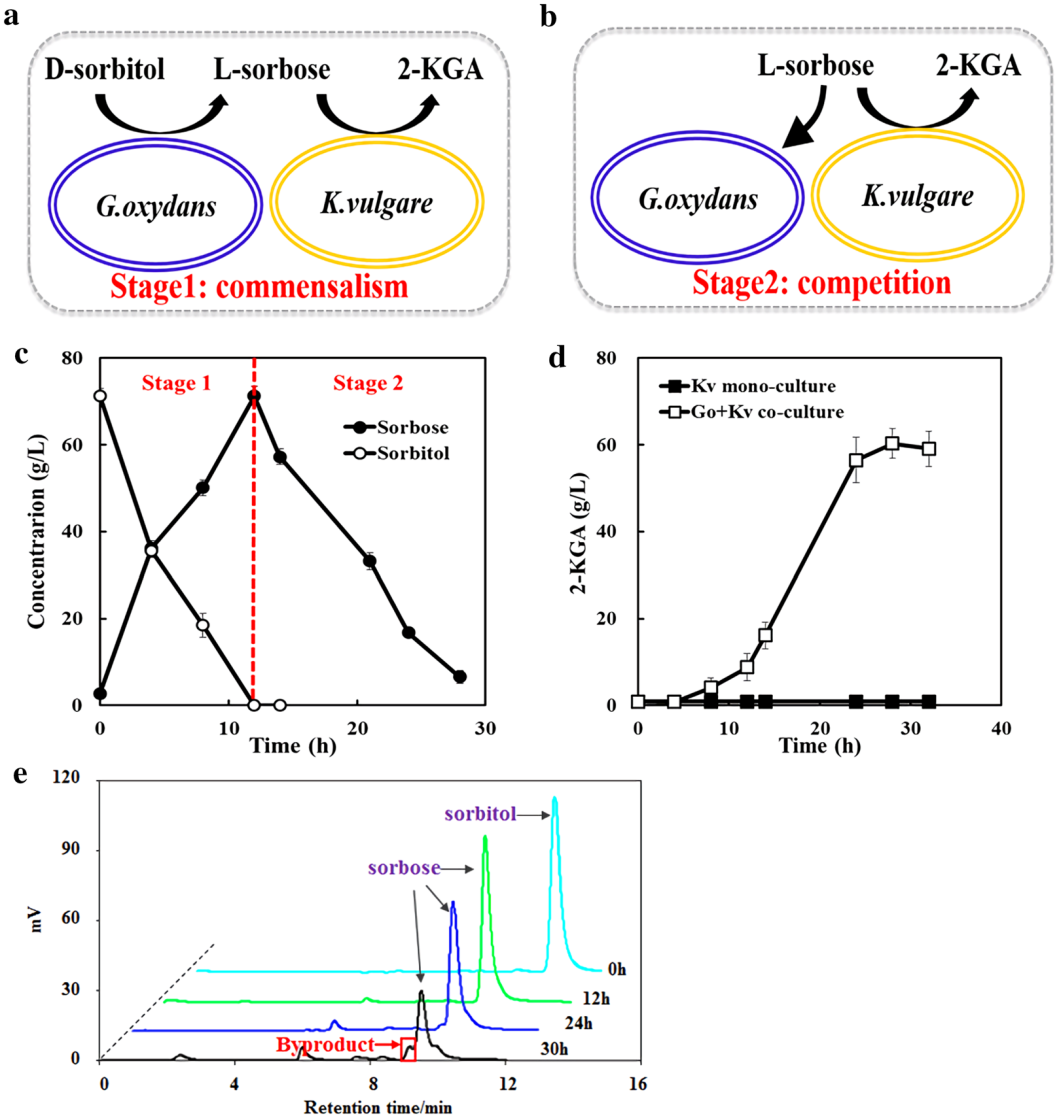
The relationship analysis of *G. oxydans*–*K. vulgare* consortium. **a** A commensalistic *G. oxydans*–*K. vulgare* consortium for production of 2-KGA within stage 1; **b** a competitive *G. oxydans*–*K. vulgare* consortium for production of 2-KGA within stage 2; **c** the division of two stages in accordance with the d-sorbitol consumption by *G. oxydans*. **d** only the co-culture produces 2-KGA; **e** chromatograms from HPLC analysis of d-sorbitol assimilation catalyzed by *G. oxydans*, the peak in the *red* box in shows the unknown byproduct produced by *G. oxydans* accompany with the sorbose assimilation

Because of the survival traits of *K. vulgare*, the companion bacterium is not confined to *Bacillus* spp., some other bacteria, such as *Xanthomonas maltophilia* [22], can also be a good partner. Considering these conditions, *Bacillus* spp. was removed in this study and a synthetic consortium of *G. oxydans*–*K. vulgare* producing 2-KGA directly from d-sorbitol was constructed (Additional file 1: Figure S2a). The relationship within this synthetic microbial consortium was analyzed for further study. The whole fermentation process of *G. oxydans* is divided into two stages on the basis of d-sorbitol assimilation. In the co-culture system, the relationship turns from the commensalism of the first stage to the competition of the second stage, in accordance with the d-sorbitol consumption by *G. oxydans* (Fig. 1a–c).

The titer of 2-KGA by this consortium was only 12.9 g/L within 36 h, and the yield was 15.0 %, which was much lower than that of the industrial two-step fermentation process (about 90 %). In order to improve the performance of this one-step fermentation process, many optimization attempts have been made, including modification of the inoculation ratio, agitation speed, and aeration rate. In this way, the titer of 2-KGA reached to 59.1 g/L within 28 h, which shorten the fermentation time by about 40 % (Additional file 1: Figure S2b). Whereas in the control experiment in which only *K. vulgare* (Fig. 1d) or *G. oxydans* (data not shown) was cultured, no 2-KGA was produced. These results showed that l-sorbose produced by *G. oxydans* diffused into *K. vulgare* cells and was subsequently oxidized. However, though the optimization of fermentation conditions indeed improved the titer and yield of 2-KGA, the natural limitation of l-sorbose consumption by *G. oxydans* in this consortium cannot be overcome without genetic modification. In this study, *G. oxydans* was cultivated in d-sorbitol seed culture medium and the composition of the culture broth from at different time points during the fermentation was measured by HPLC. We found that l-sorbose cannot be consumed until d-sorbitol was exhausted after 12 h in the mono-culture of *G. oxydans*, which matched the conclusion drawn by Soemphol et al. [23]. Then the accumulation of an unknown byproduct was detected while l-sorbose was consumed (Fig. 1e), which would reduce the 2-KGA production and make the efficiency too low to fully meet industrial requirements. In industrial fermentation, even one percent loss of carbon source will cause a significant financial burden. Therefore, we further optimized this two-strain consortium by alleviating the metabolic competition of *G. oxydans* with *K. vulgare* for sorbose, which was helpful for establishing a better homeostasis between microbes and making them work better together.

### The relationship optimization of *G. oxydans*–*K. vulgare* consortium

**Fig. 2:**
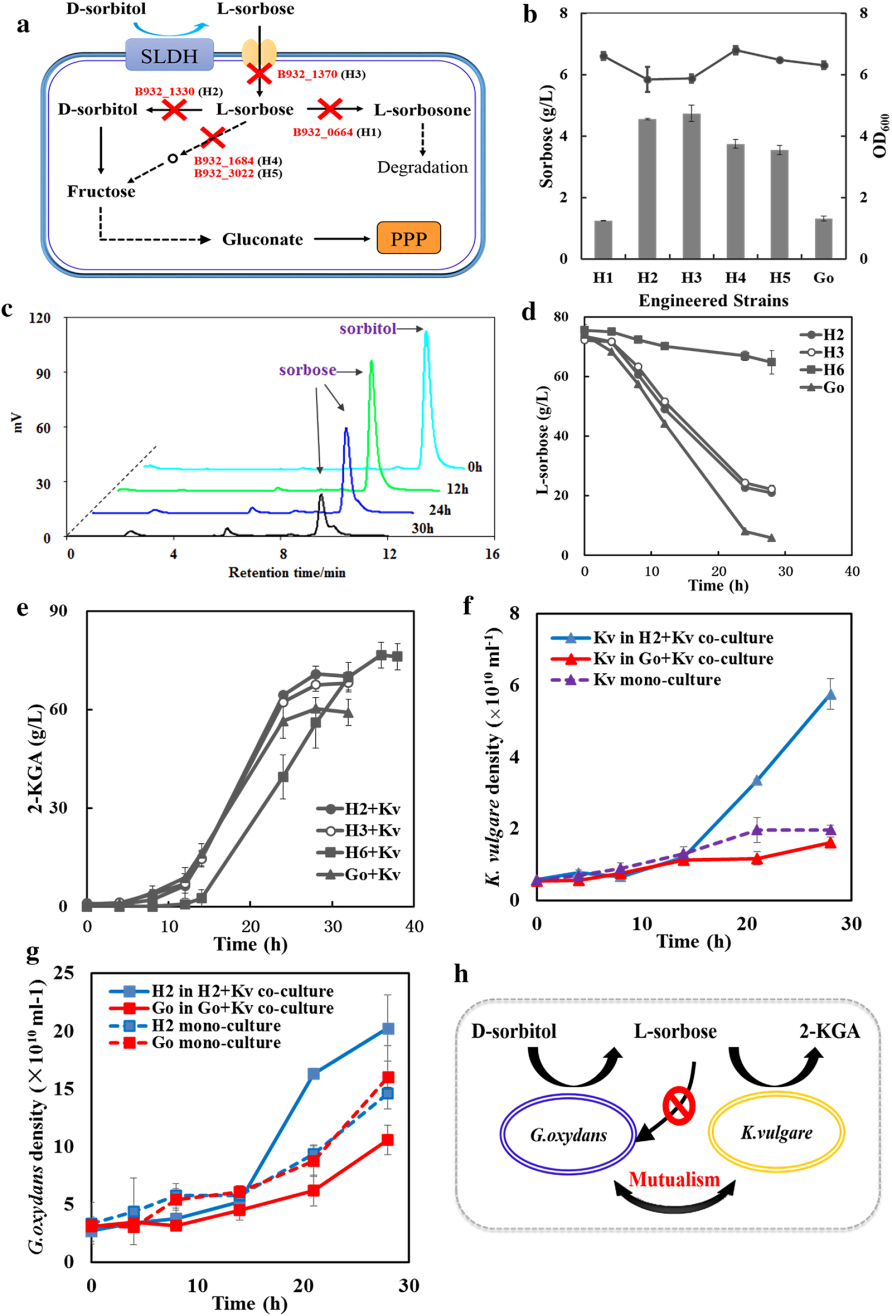
The relationship analysis of the reorganized *G. oxydans*–*K. vulgare* consortium. **a** Sorbose metabolism in *G. oxydans*, *red* words indicate the genes involved in sorbose metabolism in *G. oxydans*; **b** effect of engineered strains on l-sorbose assimilation in the flasks; **c** chromatograms from HPLC analysis of d-sorbitol assimilation catalyzed by engineered H_2_, where no byproduct can be detected with the sorbose assimilation; **d** effect of engineered strain on l-sorbose assimilation in the jar fermentors; **e** effect of engineered *G. oxydans* strains on 2-KGA accumulation in the synthetic consortium; **f** *K. vulgare* density in co- and mono-culture; **g** *G. oxydans* and engineered H_2_ density in co- and mono-culture; **h** a mutualistic *G. oxydans*–*K. vulgare* consortium for production of 2-KGA

The core process for the synthetic microbial consortium we designed in this study was the conversion of l-sorbose, the substrate for *K. vulgare* to synthesize the final product 2-KGA. After screening the genome information of *G. oxydans* [24], five relevant genes (Table 1) encoding FAD-dependent l-sorbose 1-dehydrogenase, NADPH-dependent l-sorbose reductase, and PTS system transporter subunit IIA in the l-sorbose consumption pathway were deleted, respectively. And five engineered *G. oxydans* strains, namely H_1_, H_2_, H_3_, H_4_, and H_5_ (Fig. 2a), were obtained with the method mentioned in “Strains” section. Fermentations of each engineered strains were carried out in flasks and jar fermentors to test the effect of the gene deletions. Compared with *G. oxydans*, the engineered H_2_, H_3_, H_4_, and H_5_ significantly slowed down the consumption of l-sorbose and increased the level of l-sorbose left in the broth after 30 h cultivation in l-sorbose seed culture medium in flasks (Fig. 2b). Among them, H_2_ and H_3_ were considered the most effective candidates for reducing sorbose utilization in *G. oxydans*. Consequently, a double mutant strain (H_6_) was constructed by further deletion of NADPH-dependent l-sorbose reductase (B932_1330) in H_3_ to perform better for 2-KGA production in the consortium. After that, H_2_, H_3_ and H_6_ were individually co-cultured with *K. vulgare,* forming consortia H_2_ + Kv, H_3_ + Kv and H_6_ + Kv, in the medium contained 8 % d-sorbitol as substrate. The alleviation of competition and the enhancement of mutualism were verified by the undetected byproduct (Fig. 2c), the level of remaining sorbose in the broth (Fig. 2d) and the titer of 2-KGA (Fig. 2e). As a result, H_2_, H_3_ and H_6_ enabled an 18.6, 15.2 and 29.6 % increase in the production of 2-KGA (70.1, 68.1 and 76.6 g/L) respectively compared to the primary consortium Go + Kv (59.1 g/L) after 28 or 36 h of cultivation. The relevant data of this study was compared with that of the conventional two-step fermentation (Additional file 1: Table S1). On one hand, the yield of 2-KGA was about 99 and 91 % for each stage with 8 % d-sorbitol as substrate in the two-step fermentation process. While in our study, it reached 89.7 % with the same amount substrate and shortened the fermentation time by about 25 %. On the other hand, our route eliminated the need for a second sterilization process, where the rate of equipment utilization can be significantly improved and the production cost can be notably saved.

The optimization of the relationship between the two microbes, *G. oxydans* and *K. vulgare*, was further studied. The consortia population compositions throughout the process were analyzed to validate the variation of the relationship. Figure 2f and g showed the relative density of different microbes in co- and mono-cultured systems. We found that the *K. vulgare* in the engineered consortium H_2_ + Kv also showed a better growth than that in the primary consortium Go + Kv (Fig. 2f), coupled with the higher production of 2-KGA in H_2_ + Kv. Meanwhile, the growth levels of engineered H_2_ and *G. oxydans* were similar in mono-culture. While after the introduction of *K. vulgare*, the engineered H_2_ grew much faster than the wild type since 8 h after inoculation and reached almost twice of the wild type after 28 h (Fig. 2g). In the present study, another interesting phenomenon about the initial inoculum ratio of *G. oxydans* to *K. vulgare* has been found. The inoculum ratio (%, v/v) of *G. oxydans* to *K. vulgare* was firstly set as 1:4 because of the growth defect of *K. vulgare*. Due to a low yield of 2-KGA, we then adjusted it to 4:1, which led to a great improvement in 2-KGA productivity (data not shown). This appears to be a counterintuitive finding that high ratio of inoculated *G. oxydans* was beneficial for the synthetic consortium. We speculated that because of the survival traits of *K. vulgare*, more *G. oxydans* were needed to provide more nutrients for the growth and productivity of *K. vulgare*. It was found that *G. oxydans* was the most populous consortium member throughout the whole process. However the ratio of *G. oxydans* to *K. vulgare* decreased during the fermentation in the engineered consortium, which was contrary to the original consortium Go + Kv. From this point of view, the engineered H_2_ promoted the growth and productivity of *K. vulgare* and the latter stimulated the growth of H_2_ in return. We hypothesized that there was more interaction of biomolecules or information signals between the two microbes in this mutualistic *G. oxydans*–*K. vulgare* consortium (Fig. 2h) compared with the primary competitive consortium. Hence, metabolomic analysis of the different consortium should be done for a comprehensive description of the relationship optimization between the members.

### Metabolomic analysis on the relationship optimization of *G. oxydans*–*K. vulgare* consortium

**Fig. 3:**
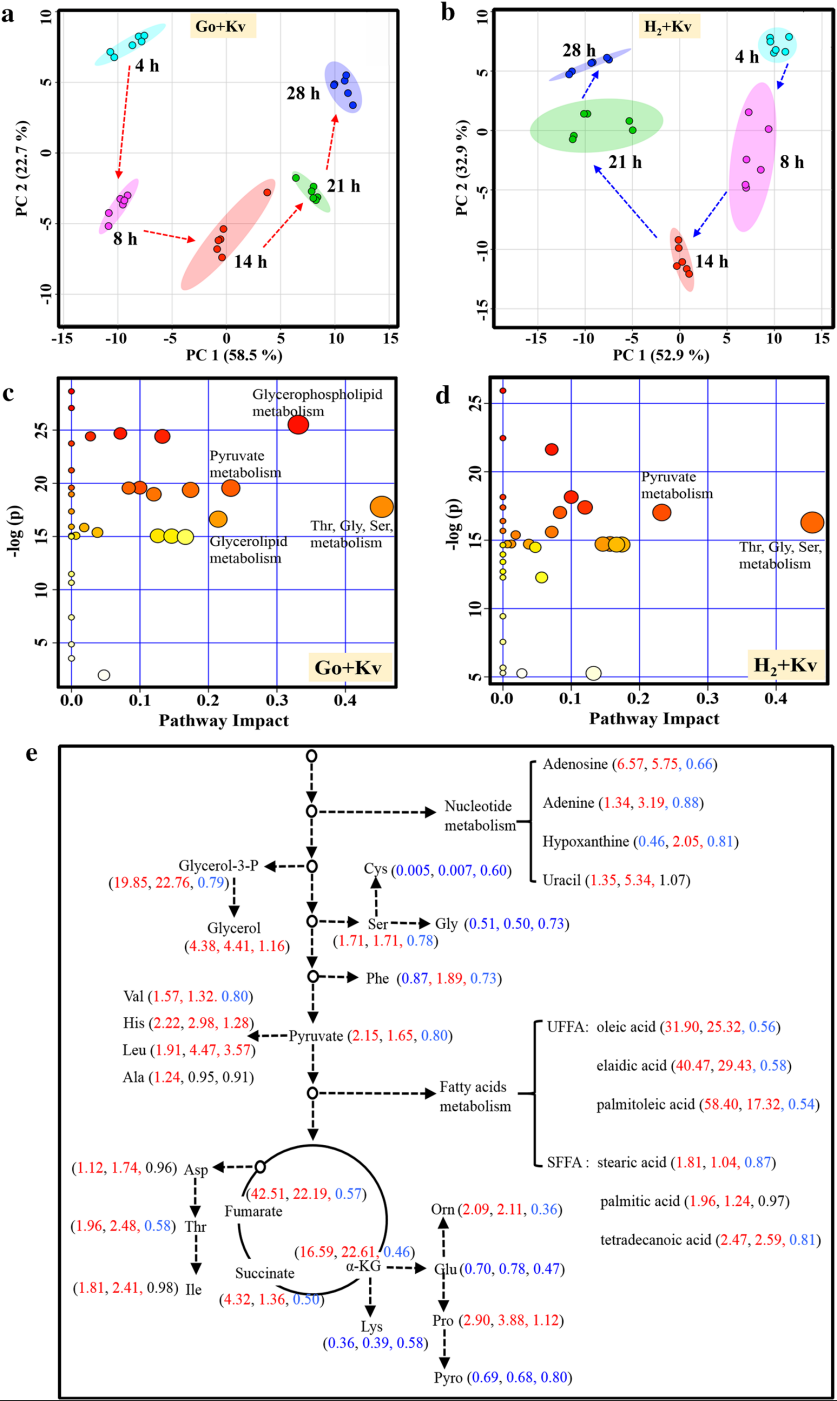
Metabolomic analysis on the relationship optimization of *G. oxydans*–*K. vulgare* consortium. **a** PCA score plot of time-series metabolomics data of Go + Kv; **b** PCA score plot of time-series metabolomics data of H_2_ + Kv; **c** Pathway enrichment analysis of consortium Go + Kv, *circles* represent all the matched pathways according to KEGG. The color and size of each circle is based on *P* values (*y-axis*) and pathway impact values (*x-axis*), respectively. The *darker* the *circle color*, the more significant and coordinated changes of metabolites in the matched metabolic pathway. The *bigger* the *circle size*, the higher the centrality of its involved metabolites. **d** Pathway enrichment analysis of consortium H_2_ + Kv; **e** Fold changes of metabolites in central carbon metabolism and related metabolism. The *numbers* in the bracket from *left* to *right* indicated the fold changes of metabolite abundance in consortium Go + Kv relative to Go, in H_2_ + Kv relative to H_2_, and in H_2_ + Kv relative to Go + Kv, respectively. *Red* numbers indicate the increased levels, and *blue* numbers indicate the decreased levels

Simplifying the sorbose metabolic pathway will affect not only itself alone, but also other related metabolic characteristics. Thus, the metabolome of the engineered consortium H_2_ + Kv was compared with the primary consortium Go + Kv to better understand the metabolic changes. It was found by PCA that the metabolomic data of the consortia Go + Kv and H_2_ + Kv at different sampling times (4, 8, 14, 21, 28 h) grouped clearly, respectively. An interesting phenomenon was that the metabolism of consortia Go + Kv and H_2_ + Kv had opposite trajectories over time (Fig. 3a, b). It indicated that the metabolic characteristics of the consortium changed after the *G. oxydans* was replaced by the engineered H_2_. Pathway enrichment analysis was then carried out on the metabolomic data of consortia Go + Kv and H_2_ + Kv (Fig. 3c, d), we found that the glycine, serine and threonine metabolism pathways and the pyruvate metabolism pathway were among the most significantly impacted in both Go + Kv and H_2_ + Kv consortia. Besides, glycerophospholipid and glycerolipid metabolism were also demonstrated significant change in the H_2_ + Kv consortium compared to Go + Kv. Furthermore, the metabolism significantly impacted in two consortia with different metabolic trajectories indicated that the relationship between the two strains in both consortia were different.

#### Improved amino acids metabolism in *G. oxydans*–*K. vulgare* consortium

**Fig. 4:**
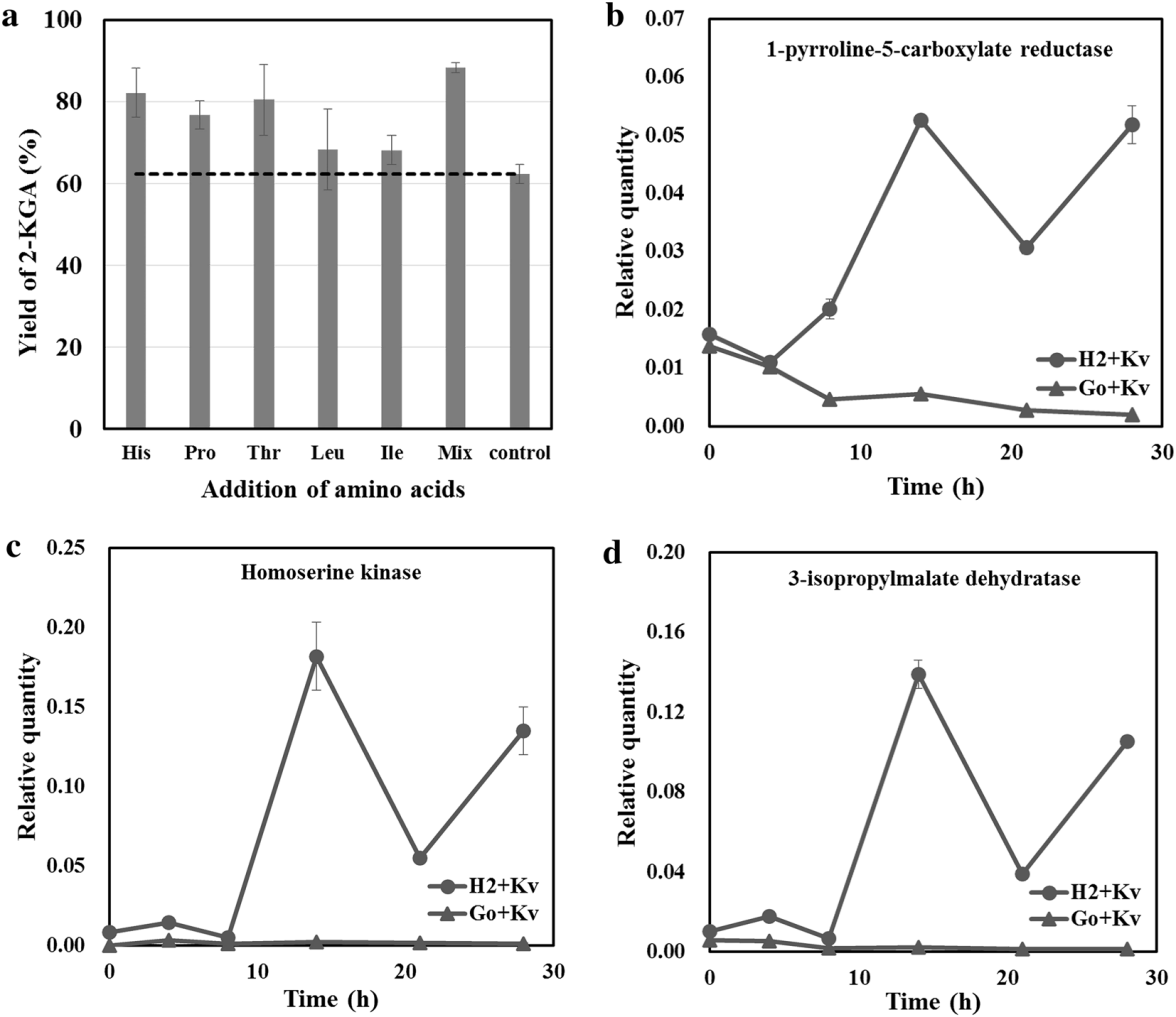
Analysis of the improved amino acids metabolism in *G. oxydans*–*K. vulgare* consortium. **a** Effect of adding amino acids on the synthetic consortium in the flask; Transcriptional expression level of **b** 1-pyrroline-5-carboxylate reductase (EC 1.5.1.2); **c** Homoserine kinase (EC 2.7.1.39); **d** 3-isopropylmalate dehydratase (EC 4.2.1.33) in co-cultured systems. All the data were normalized to 16S rDNA of each species

A complete understanding of microbial metabolism should extend from the properties of individual strain in pure culture to the combinatorial interactions supported by complex communities. Metabolic levels of *G. oxydans* in monoculture were compared with those in the consortium of *G. oxydans* and *K. vulgare* to better understand the metabolic effects by *K. vulgare*, and the metabolic interaction between *G. oxydans* and *K. vulgare* in the synthetic microbial consortium. Our metabolomics analysis showed that the metabolism of the TCA cycle, amino acids, purines and free fatty acids were all significantly affected by the introduction of *K. vulgare* to the fermentation of sorbitol by *G. oxydans* or H_2_. The variations of these metabolites of the consortium were compared with those of Go or H_2_, and the fold changes of metabolites in the consortium after engineering relative to those in primary one are shown in Fig. 3e. In this study, when part of *G. oxydans* was replaced by *K. vulgare* for fermentation, the levels of most intracellular amino acids were found to change a lot. It was reported by Liu et al. [25] that the genes contributing to the de novo biosynthesis of histidine (His), glycine (Gly), lysine (Lys), proline (Pro), threonine (Thr), methionine (Met), leucine (Leu), and isoleucine (Ile) were absent in *K. vulgare*. Thus, it was supposed that the amino acids levels in the consortium would be lower than that in *G. oxydans* monoculture. However, five of the eight deficient amino acids including His, Pro, Thr, Leu and Ile in the consortium represented higher levels in synthetic consortium, suggesting that *G. oxydans* synthetized more of these amino acids, allowing for the better growth and production of *K. vulgare*. As a consequence, we speculated that the proper supplement of these amino acids would promote better growth and production of *K. vulgare*, which improved the interaction of two strains during fermentation. In order to prove this hypothesis, we investigated the effect of these amino acids on the productivity of the consortium. These five amino acids were added into the fermentation medium individually and they did enhance the ability of 2-KGA productivity to some extent as expected (Fig. 4a). Next, a mixture of His, Pro, Thr, Leu and Ile, with a final concentration of 0.7, 0.3, 0.5, 0.1, 0.5 g/L, respectively, was added to the consortium of *G. oxydans* and *K. vulgare*. With the addition of these amino acids, the yield of 2-KGA in flask cultures after 36 h of cultivation reached 88.3 %, enjoying a 41.8 % increase compared to the original consortium (62.3 %) with no addition of amino acids (Fig. 4a). In addition, the transcriptional expression level of histidinol-phosphatase, 1-pyrroline-5-carboxylate reductase, homoserine kinase and 3-isopropylmalate dehydratase in co- and mono-cultured systems at different sampling time points was evaluated by qPCR for further evidence. It was found that 1-pyrroline-5-carboxylate reductase, homoserine kinase and 3-isopropylmalate dehydratase in synthetic consortium enjoyed a similar tendency (Fig. 4b–d). The transcriptional expression of these genes in H_2_ + Kv consortium showed a highest level at the early point of exponential phase (14 h) as expected, which was in accordance with the tendency of growth and productivity of *K. vulgare*. However, the levels of these genes were much lower in Go + Kv consortium and no expression could be detected in the mono-culture of *K. vulgare*. From this point of view, it proved that with the stimulation of *K. vulgare*, *G. oxydans* synthetized more of these amino acids for better growth and production of *K. vulgare*.

#### Improved purines metabolism in *G. oxydans*–*K. vulgare* consortium

**Fig. 5:**
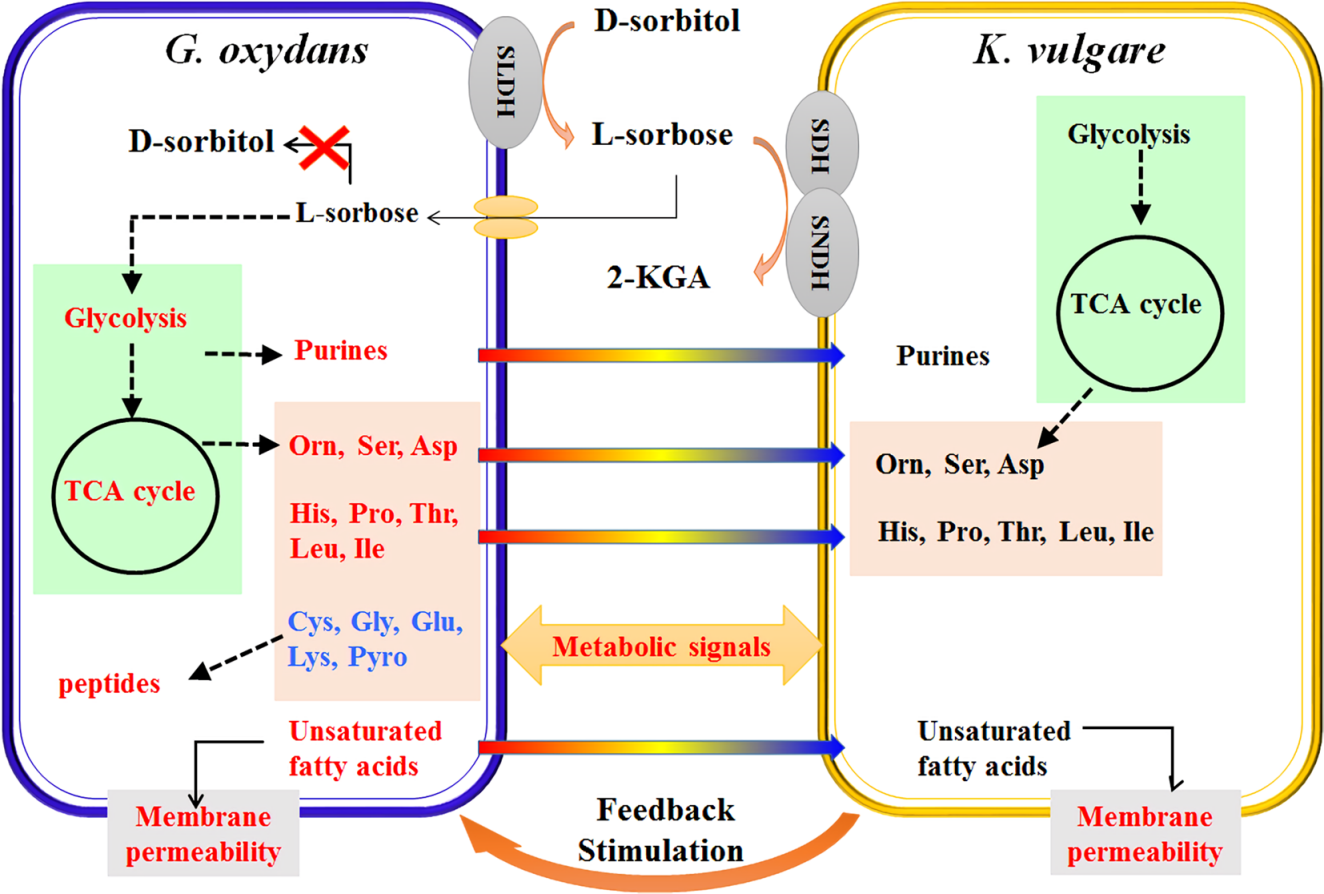
Schematic of the interaction mechanism between *G. oxydans* and *K. vulgare* in the synthetic consortium. *Red words* and *blue words* indicate the induced and inhibited metabolites or pathways due to the interaction of *G. oxydans* and *K. vulgare,* respectively; *red arrow* indicate the enhancement of cell–cell interaction after deletion of sorbose metabolism related genes

In addition to amino acid biosynthesis deficiencies, *K. vulgare* was reported to be insufficient in purine nucleotide biosynthesis [26, 27]. Our previous study also found that supplement of purines did have certain positive effects on the cell growth and the 2-KGA productivity of *K. vulgare*. [12]. In this study, adenine and adenosine contents were both higher in consortia Go + Kv and H_2_ + Kv when compared with mono-cultured *G. oxydans* and H_2_, respectively (Fig. 3e). We also found that adenine and adenosine were undetected in mono-cultured *K. vulgare*. This suggests that *G. oxydans* provides these purines to *K. vulgare* when co-cultured, while in return, *K. vulgare* stimulates the biosynthesis of purines in *G. oxydans*. In addition, the levels of these purines decreased after engineering, which indicated the gene deletion did affect the biosynthesis of purines. However, the increase of the purines levels in co-cultured H_2_ + Kv compared to the mono-cultured H_2_ was larger than that in co-cultured Go + Kv comparing to *G. oxydans*, which suggested that co-culturing with *K. vulgare* promoted the biosynthesis of purines more in the engineered *G. oxydans* than that in the wild type. Therefore, on one hand, we suggested that *G. oxydans* provided substrates and nutrients for *K. vulgare*. On the other hand, *K. vulgare* gave some feedback to stimulate the synthesis of nutrients in *G. oxydans* (Fig. 5).

#### Improved fatty acids metabolism in *G. oxydans*–*K. vulgare* consortium

In this study, all the detected free fatty acids represented higher levels in consortium samples than that in mono-cultured *G. oxydans* samples, especially the unsaturated fatty acids including oleic acid (18:1), elaidic acid (18:1), and palmitelaidic acid (16:1). All of these three fatty acids were presented at over 20-fold higher levels in both consortia compared to monoculture. Additionally, their levels in consortium Go + Kv were higher than those in H_2_ + Kv, respectively (Fig. 3e). It was reported that the increased unsaturated fatty acid level facilitated the stress defense [28]. Thus, we speculated that *G. oxydans* might be subjected to several stresses after co-cultured with *K. vulgare*, such as the changed growth environment caused by the metabolites secreted by *K. vulgare*. More unsaturated fatty acids were synthesized by *G. oxydans* to respond to the pressure of the co-culture conditions. Compared to Go + Kv, the lower levels of unsaturated fatty acids in consortium H_2_ + Kv suggested that the engineered H_2_ possessed preferable adaptability to the environment co-culture with *K. vulgare*. On the other hand, more unsaturated fatty acids would increase the cell membrane fluidity and permeability under unfavorable conditions by affecting the plasma membrane integrity, fluidity and function [29]. The dramatic increase in the levels of these unsaturated fatty acids may indicate that cells in this consortium increased their membrane permeability for exchanging more nutrients, which would promote the interaction between two strains.

## Conclusions

In this study, a synthetic consortium for one-step vitamin C fermentation was reorganized with *G. oxydans* and *K.vulgare.* Further optimization was carried out to alleviate the competition for sorbose of *G. oxydans* with *K.vulgare*. The yield of 2-KGA of this consortium reached 89.7 % within 36 h, which is comparable to the conventional two-step fermentation. The metabolic interaction between the strains was further investigated by metabolomics, which verified the enhancement of the mutualism between the microbes and gave us a better understanding of the synthetic consortium.

## Additional file

10.1186/s12934-016-0418-6 Comparison of one-step and two-step fermentation process. **Figure S1.** Double-crossover homologous recombination schematic diagram. **Figure S2.** A new one-step fermentation route for production of 2-KGA. **a** Redesign of the conventional industrial fermentation route for one-step 2-KGA production; **b** 2-KGA accumulation in the synthetic consortium of *G. oxydans*–*K. vulgare*. *Circle* indicates the inoculation ratio of *G. oxydans *and *K. vulgare *was 1:4, the agitation speed was 400 rpm, the aeration rate was1.0 vvm; *triangle* indicates the inoculation ratio of *G. oxydans* and *K. vulgare* was 1:2, the agitation speed was 400 rpm, the aeration rate was1.0 vvm; *square* indicates the inoculation ratio of *G. oxydans* and *K. vulgare* was 4:1, the agitation speed was 500 rpm, the aeration rate was1.5 vvm.
