# Supplementary material for: Reorganization of a synthetic microbial consortium for one-step vitamin C fermentation
Source: Microb Cell Fact. 2016 Jan 25;15:21. doi: 10.1186/s12934-016-0418-6 (PMC4727326; doi:10.1186/s12934-016-0418-6)
Supplement: Supplementary file 1 — 10.1186/s12934-016-0418-6 Comparison of one-step and two-step fermentation process. Figure S1. Double-crossover homologous recombination schematic diagram. Figure S2. A new one-step fermentation route for production of 2-KGA. a Redesign of the conventional industrial fermentation route for one-step 2-KGA production; b 2-KGA accumulation in the synthetic consortium of G. oxydans–K. vulgare. Circle indicates the inoculation ratio of G. oxydans and K. vulgare was 1:4, the agitation speed was 400 rpm, the aeration rate was1.0 vvm; triangle indicates the inoculation ratio of G. oxydans and K. vulgare was 1:2, the agitation speed was 400 rpm, the aeration rate was1.0 vvm; square indicates the inoculation ratio of G. oxydans and K. vulgare was 4:1, the agitation speed was 500 rpm, the aeration rate was1.5 vvm. [file 12934_2016_418_MOESM1_ESM.doc]

**Supplementary material**

**Reorganization of a synthetic microbial consortium for one-step vitamin C fermentation**

En-Xu Wang^a,b^, Ming-Zhu Ding^a,b^*, Qian Ma^a,b^, Xiu-Tao Dong^a,b^, Ying-Jin Yuan^a,b^

a: Key Laboratory of Systems Bioengineering (Ministry of Education), School of Chemical Engineering and Technology, Tianjin University, Tianjin, 300072, PR China

b: SynBio Research Platform, Collaborative Innovation Center of Chemical Science and Engineering (Tianjin), Tianjin University, Tianjin, 300072, PR China

*Corresponding author: Ming-Zhu Ding (Email: mzding@tju.edu.cn, Tel: 86-22-60973987，Postal address: No. 92, Weijin Road, Nankai District, Tianjin, 300072, PR China)

Table S1 Comparison of one-step and two-step fermentation process

|  | **Two-step fermentation** | **One-step fermentation** |
| --- | --- | --- |
| Substrate | 8% D-sorbitol | 8% D-sorbitol |
| Yield (mol/mol) | About 99% and 91% for each stage respectively, totally 90%. | 89.7% |
| Fermentation Cycle | About 12 h and 36 h for each stage, totally 48 h. | About 36 h |
| Sterilization | Twice | Once |
| Equipment Capacity | Low | High |


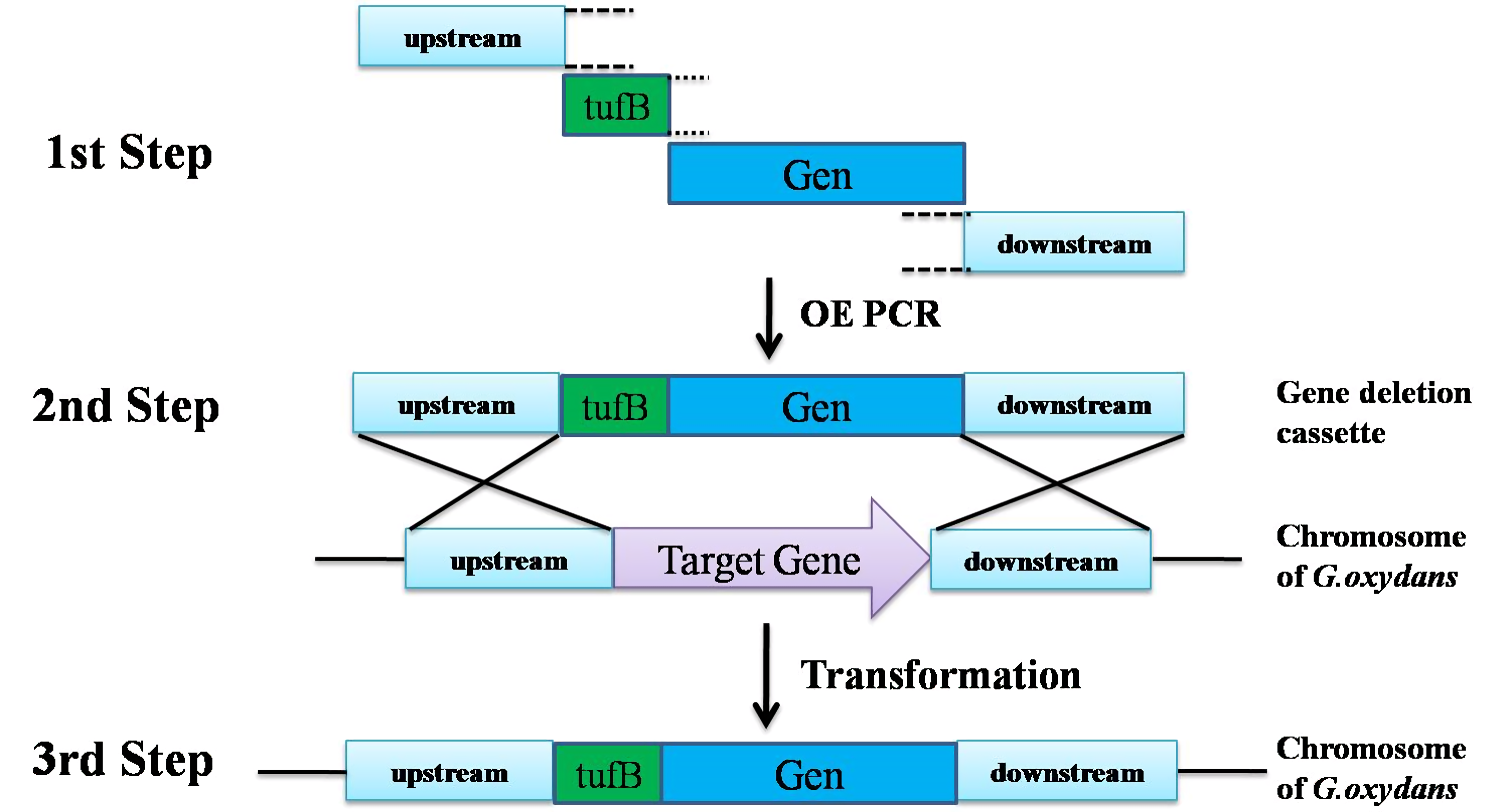


Fig. S1 Double-crossover homologous recombination schematic diagram


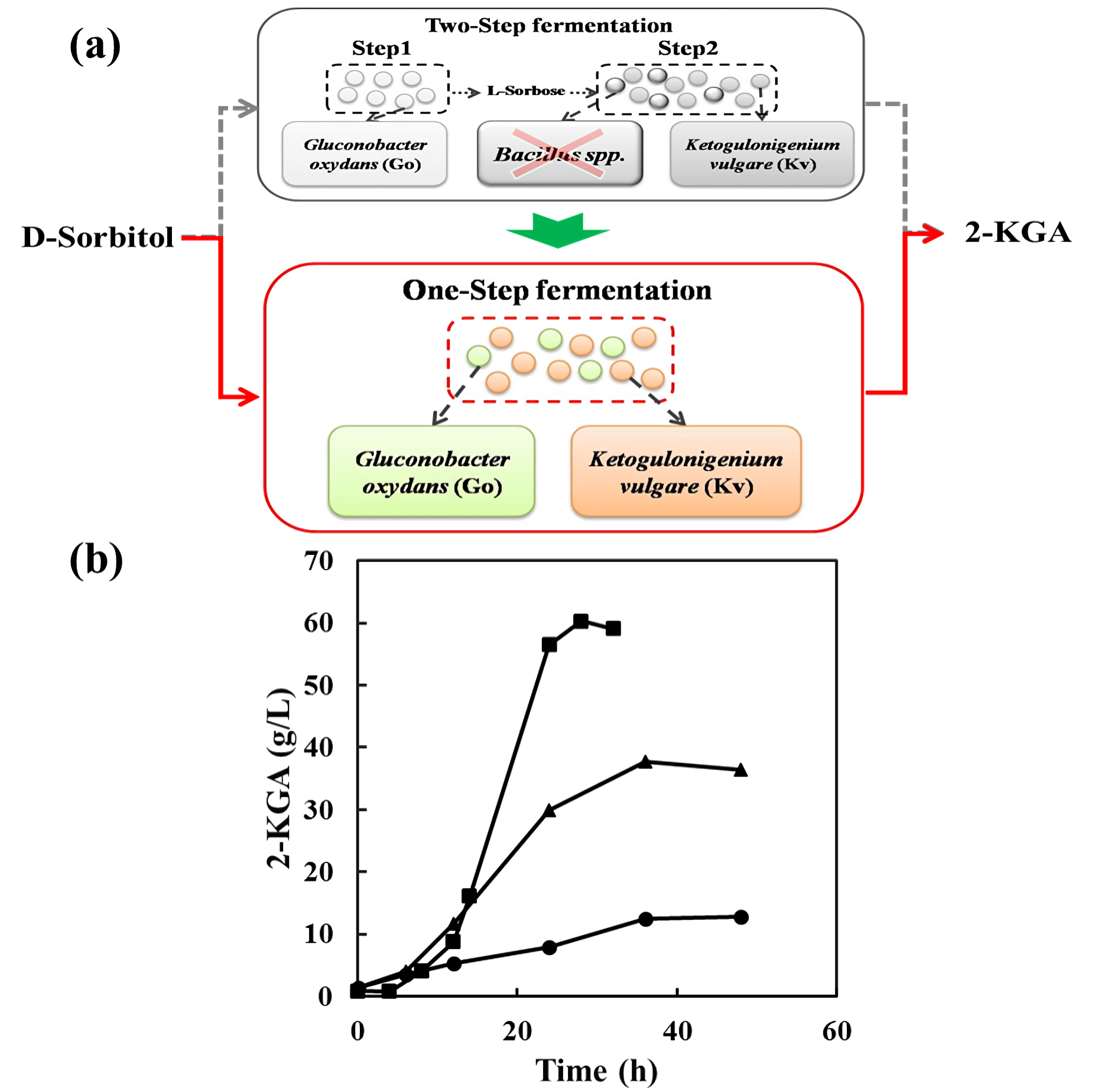


Fig. S2 A new one-step fermentation route for production of 2-KGA. (a) Redesign of the conventional industrial fermentation route for one-step 2-KGA production; (b) 2-KGA accumulation in the synthetic consortium of *G. oxydans- K. vulgare*. “circle” indicates the inoculation ratio of *G. oxydans* and *K. vulgare* was 1:4, the agitation speed was 400 rpm, the aeration rate was1.0 vvm; “triangle” indicates the inoculation ratio of *G. oxydans* and *K. vulgare* was 1:2, the agitation speed was 400 rpm, the aeration rate was1.0 vvm; “square” indicates the inoculation ratio of *G. oxydans* and *K. vulgare* was 4:1, the agitation speed was 500 rpm, the aeration rate was1.5 vvm.
